# Supplementary material for: Socioeconomic position is associated with N-terminal pro-brain natriuretic peptide (NT-proBNP)—Results of the population-based Heinz Nixdorf Recall study
Source: PLoS One. 2021 Aug 20;16(8):e0255786. doi: 10.1371/journal.pone.0255786 (PMC8378685; doi:10.1371/journal.pone.0255786)
Supplement: S1 Table — (DOCX) [file pone.0255786.s001.docx]

**S1 Table.** Characteristics of the sensitivity analysis study population (i.e., excluding participants with prevalent coronary heart disease and stroke) stratified by sex (mv: number of missing values).

|  | **All** | **Women** | **Men** |
| --- | --- | --- | --- |
| **N** (%) | 4169 (100%) | 2184 (52.4%) | 1985 (47.6%) |
| **Age** (years) | 59.2 ± 7.8 | 59.3 ± 7.8 | 59.0 ± 7.7 |
| **Income** (EURO/month) mv=271 | 1449.0 (1108; 1897) | 1406.0 (959; 1875) | 1520.0 (1150; 2173) |
| **Education** (years of training) mv=9 |  |  |  |
| ≤ 10 (low) | 471 (11.3%) | 374 (17.1%) | 97 (4.9%) |
| 11 – 13 | 2307 (55.5%) | 1383 (63.4%) | 924 (46.7%) |
| 14 – 17 | 930 (22.4%) | 254 (11.6%) | 676 (34.2%) |
| ≥ 18 (high) | 452 (10.9%) | 171 (7.8%) | 281 (14.2%) |
| **NT-proBNP** (pg/ml) | 68.0 (37; 123) | 84.5 (52; 142) | 49.0 (28; 94) |
| **BMI** (kg/m²) mv=17 | 27.8 ± 4.7 | 27.5 ± 5.2 | 28.1 ± 4.0 |
| **Systolic blood pressure** (mmHg) | 132.7 ± 20.8 | 127.9 ± 20.9 | 138.0 ± 19.4 |
| **Diastolic blood pressure (**mmHg) mv=6 | 81.6 ± 10.9 | 78.9 ± 10.5 | 84.5 ± 10.5 |
| **Anti-hypertensive medication** | 1303 (31.3%) | 712 (32.6%) | 591 (29.8%) |
| **Total cholesterol** (mg/dl) | 231.1 ± 38.8 | 234.1 ± 39.4 | 227.9 ± 38.0 |
| **LDL cholesterol** (mg/dl) mv=12 | 147.1 ± 36.1 | 146.5 ± 36.8 | 147.7 ± 35.4 |
| **HDL cholesterol** (mg/dl) mv=1 | 58.9 ± 17.2 | 65.4 ± 16.8 | 51.7 ± 14.5 |
| **Lipid-lowering medication** mv=257 | 358 (9.2%) | 204 (9.9%) | 154 (8.3%) |
| **Diabetes mellitus** | 494 (11.8%) | 193 (8.8%) | 301 (15.2%) |
| **Current Smoking** mv=3 | 973 (23.4%) | 467 (21.4%) | 506 (25.5%) |
