# Supplementary material for: Socioeconomic position is associated with N-terminal pro-brain natriuretic peptide (NT-proBNP)—Results of the population-based Heinz Nixdorf Recall study
Source: PLoS One. 2021 Aug 20;16(8):e0255786. doi: 10.1371/journal.pone.0255786 (PMC8378685; doi:10.1371/journal.pone.0255786)
Supplement: S2 Table — (DOCX) [file pone.0255786.s002.docx]

**S2 Table.** Effect size estimates as percentage change in NT‑proBNP per 1000€ income/month and 95% confidence intervals (95%‑CI) for the main analysis population and stratified by sex.

|  | | **%-Change (95%-Confidence Interval)** | | | | | | | | | | | |
| --- | --- | --- | --- | --- | --- | --- | --- | --- | --- | --- | --- | --- | --- |
| **Model, subgroup** | **N** | **Intercept** | **Income (per 1000€)** | **Age** | **Sex [female]** | **Diabetes mellitus [yes]** | **Systolic blood pressure** | **HDL Cholesterol** | **LDL Cholesterol** | **Anti-hypertensive medication** | **Lipid-lowering medication** | **BMI** | **Current smoking** |
| **Model 1, all** | 4305 | 375.72 (282.02 ; 492.40) | -6.47 (-9.91 ; -2.91) | 4.56 (4.21 ; 4.91) | 45.36 (37.92 ; 53.20) |  |  |  |  |  |  |  |  |
| **Model 2, all** | 4013 | 710.78 (474.46 ; 1044.32) | -5.75 (-9.25 ; -2.13) | 3.75 (3.36 ; 4.13) | 50.73 (41.95 ; 60.04) | -5.74 (-12.95 ; 2.07) | 0.38 (0.24 ; 0.52) | 0.05 (-0.13 ; 0.23) | -0.33 (-0.41 ; -0.26) | 46.28 (37.72 ; 55.38) | 4.6 (-3.59 ; 13.49) | -1.07 (-1.70 ; -0.44) | 8.02 (1.26 ; 15.23) |
| **Model 1, men** | 2208 | 124.59 (63.47 ; 208.57) | -8.43 (-13.21 ; -3.38) | 5.95 (5.43 ; 6.47) |  |  |  |  |  |  |  |  |  |
| **Model 2, men** | 2042 | 183.49 (65.05 ; 386.90) | -7.36 (-12.24 ; -2.21) | 4.74 (4.18 ; 5.31) |  | -0.89 (-10.81 ; 10.13) | 0.58 (0.36 ; 0.79) | 0.09 (-0.20 ; 0.38) | -0.27 (-0.39 ; -0.16) | 51.19 (38.45 ; 65.10) | 20.75 (7.50 ; 35.63) | -0.74 (-1.79 ; 0.33) | 9.68 (0.05 ; 20.24) |
| **Model 1, women** | 2097 | 1417.54 (1038.57 ; 1922.64) | -5.1 (-9.81 ; -0.13) | 3.15 (2.70 ; 3.61) |  |  |  |  |  |  |  |  |  |
| **Model 2, women** | 1971 | 2611.88 (1647.72 ; 4107.94) | -4.95 (-9.71 ; 0.07) | 2.74 (2.23 ; 3.25) |  | -15.14 (-24.88 ; -4.14) | 0.26 (0.08 ; 0.44) | -0.03 (-0.25 ; 0.19) | -0.32 (-0.41 ; -0.22) | 40.33 (29.43 ; 52.14) | -9.37 (-19.05 ; 1.47) | -1.04 (-1.78 ; -0.29) | 4.73 (-4.19 ; 14.47) |

# Model 1: adjusted for age, (sex); Model 2: adjusted for age, (sex), systolic blood pressure, HDL cholesterol, LDL cholesterol, diabetes, anti‑hypertensive medication, lipid‑lowering medication, BMI and current smoking.
