# Supplementary material for: Socioeconomic position is associated with N-terminal pro-brain natriuretic peptide (NT-proBNP)—Results of the population-based Heinz Nixdorf Recall study
Source: PLoS One. 2021 Aug 20;16(8):e0255786. doi: 10.1371/journal.pone.0255786 (PMC8378685; doi:10.1371/journal.pone.0255786)
Supplement: S3 Table — (DOCX) [file pone.0255786.s003.docx]

**S3 Table.** Effect size estimates as percentage change in NT‑proBNP per 1000€ income/month and 95% confidence intervals (95%‑CI) for the main analysis population adjusted for age, sex and separately for one single cardiovascular risk factor.

| **CVD risk factor** | **N** | **%-Change per 1000€ income/month** | **95%-CI** |
| --- | --- | --- | --- |
| **BMI** | 4285 | -6.47 | -9.92; -2.88 |
| **Systolic blood pressure** | 4301 | -5.95 | -9.41; -2.37 |
| **Diastolic blood pressure** | 4302 | -6.51 | -9.95; -2.94 |
| **Anti-hypertensive medication** | 4289 | -5.11 | -8.53; -1.56 |
| **Total cholesterol** | 4305 | -6.72 | -10.11; -3.21 |
| **LDL cholesterol** | 4293 | -7.17 | -10.55; -3.67 |
| **HDL cholesterol** | 4304 | -6.51 | -9.94; -2.94 |
| **Lipid-lowering medication** | 4045 | -6.69 | -10.22; -3.02 |
| **Diabetes mellitus** | 4305 | -6.41 | -9.84; -2.84 |
| **Current Smoking** | 4305 | -6.29 | -9.73; -2.71 |
