# Supplementary material for: Socioeconomic position is associated with N-terminal pro-brain natriuretic peptide (NT-proBNP)—Results of the population-based Heinz Nixdorf Recall study
Source: PLoS One. 2021 Aug 20;16(8):e0255786. doi: 10.1371/journal.pone.0255786 (PMC8378685; doi:10.1371/journal.pone.0255786)
Supplement: S4 Table — (DOCX) [file pone.0255786.s004.docx]

**S4** **Table.** Effect size estimates as percentage change in NT‑proBNP per year of education and 95% confidence intervals (95%‑CI) for the main analysis population and stratified by sex.

|  | | **%-Change (95%-Confidence Interval)** | | | | | | | | | | | |
| --- | --- | --- | --- | --- | --- | --- | --- | --- | --- | --- | --- | --- | --- |
| **Model, subgroup** | **N** | **Intercept** | **Education (per year)** | **Age** | **Sex [female]** | **Diabetes mellitus [yes]** | **Systolic blood pressure** | **HDL Cholesterol** | **LDL Cholesterol** | **Anti-hypertensive medication** | **Lipid-lowering medication** | **BMI** | **Current smoking** |
| Model 1, all | 4585 | 362.69 (249.08 ; 513.28) | -0.8 (-1.92 ; 0.32) | 4.63 (4.29 ; 4.97) | 45.18 (37.66 ; 53.11) |  |  |  |  |  |  |  |  |
| Model 2, all | 4274 | 702.05 (440.25 ; 1090.71) | -0.85 (-1.99 ; 0.30) | 3.83 (3.45 ; 4.20) | 49.47 (40.66 ; 58.84) | -5.79 (-12.79 ; 1.78) | 0.37 (0.24 ; 0.51) | 0.06 (-0.12 ; 0.24) | -0.34 (-0.41 ; -0.27) | 46.87 (38.55 ; 55.68) | 4.33 (-3.63 ; 12.94) | -1.02 (-1.62 ; -0.41) | 9.07 (2.47 ; 16.09) |
| Model 1, men | 2295 | 132.61 (57.24 ; 244.10) | -1.63 (-3.23 ; -0.00) | 6.06 (5.55 ; 6.58) |  |  |  |  |  |  |  |  |  |
| Model 2, men | 2125 | 198.96 (63.36 ; 447.12) | -1.44 (-3.08 ; 0.23) | 4.89 (4.34 ; 5.45) |  | -0.24 (-10.04 ; 10.63) | 0.53 (0.32 ; 0.74) | 0.16 (-0.13 ; 0.45) | -0.28 (-0.39 ; -0.17) | 51.49 (38.93 ; 65.20) | 18.75 (5.90 ; 33.16) | -0.75 (-1.79 ; 0.29) | 10.05 (0.50 ; 20.52) |
| Model 1, women | 2290 | 1493.91 (990.92 ; 2228.81) | -1.04 (-2.56 ; 0.50) | 3.18 (2.73 ; 3.63) |  |  |  |  |  |  |  |  |  |
| Model 2, women | 2149 | 2897.64 (1708.85 ; 4867.71) | -1.24 (-2.79 ; 0.33) | 2.71 (2.21 ; 3.22) |  | -15.87 (-25.14 ; -5.44) | 0.28 (0.11 ; 0.45) | -0.06 (-0.27 ; 0.15) | -0.32 (-0.41 ; -0.23) | 41.61 (31.14 ; 52.90) | -8.04 (-17.50 ; 2.51) | -1.02 (-1.73 ; -0.31) | 5.49 (-3.03 ; 14.76) |

Model 1: adjusted for age, (sex); model 2: adjusted for age, (sex), systolic blood pressure, HDL cholesterol, LDL cholesterol, diabetes, anti‑hypertensive medication, lipid‑lowering medication, BMI and current smoking.
