# Supplementary material for: Socioeconomic position is associated with N-terminal pro-brain natriuretic peptide (NT-proBNP)—Results of the population-based Heinz Nixdorf Recall study
Source: PLoS One. 2021 Aug 20;16(8):e0255786. doi: 10.1371/journal.pone.0255786 (PMC8378685; doi:10.1371/journal.pone.0255786)
Supplement: S5 Table — (DOCX) [file pone.0255786.s005.docx]

**S5 Table**. Effect size estimates as percentage change in NT‑proBNP per year of education and 95% confidence intervals (95%‑CI) for the main analysis population adjusted for age, sex and separately for one single cardiovascular risk factor.

| **CVD risk factors** | **N** | **%-Change per year of education** | **95%-CI** |
| --- | --- | --- | --- |
| **BMI** | 4563 | -0.85 | -1.97; 0.29 |
| **Systolic blood pressure** | 4580 | -0.60 | -1.71; 0.53 |
| **Diastolic blood pressure** | 4581 | -0.79 | -1.91; 0.34 |
| **Anti-hypertensive medication** | 4569 | -0.64 | -1.73; 0.47 |
| **Total cholesterol** | 4585 | -0.99 | -2.09; 0.12 |
| **LDL cholesterol** | 4572 | -1.10 | -2.20; 0.01 |
| **HDL cholesterol** | 4584 | -0.81 | -1.92; 0.32 |
| **Lipid-lowering medication** | 4309 | -0.87 | -2.03; 0.30 |
| **Diabetes mellitus** | 4585 | -0.77 | -1.88; 0.36 |
| **Current Smoking** | 4585 | -0.69 | -1.81; 0.44 |
