# Supplementary material for: Socioeconomic position is associated with N-terminal pro-brain natriuretic peptide (NT-proBNP)—Results of the population-based Heinz Nixdorf Recall study
Source: PLoS One. 2021 Aug 20;16(8):e0255786. doi: 10.1371/journal.pone.0255786 (PMC8378685; doi:10.1371/journal.pone.0255786)
Supplement: S6 Table — (DOCX) [file pone.0255786.s006.docx]

**S6 Table.** Effect size estimates as percentage change in NT-proBNP per 1000€ income/month and 95% confidence intervals (95%-CI) for the analysis population after excluding participants with prevalent coronary heart disease and stroke and stratified by sex.

| **All** | | | |
| --- | --- | --- | --- |
| **Model** | **N** | **%-Change** | **95%-CI** |
| **Model 1** | 3898 | -5.70 | -9.13; -2.14 |
| **Model 2** | 3636 | -5.09 | -8.59; -1.45 |
| **Men** | | | |
| **Model** | **N** | **%-Change** | **95%-CI** |
| **Model 1** | 1905 | -8.10 | -12.88; -3.06 |
| **Model 2** | 1761 | -6.73 | -11.67; -1.52 |
| **Women** | | | |
| **Model** | **N** | **%-Change** | **95%-CI** |
| **Model 1** | 1993 | -3.64 | -8.43; 1.40 |
| **Model 2** | 1874 | -4.01 | -8.82; 1.07 |
| Model 1: adjusted for age, (sex); model 2: adjusted for age, (sex), systolic blood pressure, HDL cholesterol, LDL cholesterol, diabetes, anti-hypertensive medication, lipid-lowering medication, BMI and current smoking. | | | |
