# Supplementary material for: Socioeconomic position is associated with N-terminal pro-brain natriuretic peptide (NT-proBNP)—Results of the population-based Heinz Nixdorf Recall study
Source: PLoS One. 2021 Aug 20;16(8):e0255786. doi: 10.1371/journal.pone.0255786 (PMC8378685; doi:10.1371/journal.pone.0255786)
Supplement: S7 Table — (DOCX) [file pone.0255786.s007.docx]

**S7 Table.** Effect size estimates as percentage change in NT-proBNP per year of education and 95% confidence intervals (95%-CI) for the analysis population after excluding participants with prevalent coronary heart disease and stroke and stratified by sex.

| **All** | | | |
| --- | --- | --- | --- |
| **Model** | **N** | **%-Change** | **95%-CI** |
| **Model 1** | 4160 | -0.84 | -1.95; 0.27 |
| **Model 2** | 3879 | -0.80 | -1.94; 0.35 |
| **Men** | | | |
| **Model** | **N** | **%-Change** | **95%-CI** |
| **Model 1** | 1978 | -1.66 | -3.25; -0.03 |
| **Model 2** | 1830 | -1.08 | -2.75; 0.62 |
| **Women** | | | |
| **Model** | **N** | **%-Change** | **95%-CI** |
| **Model 1** | 2182 | -0.76 | -2.28; 0.78 |
| **Model 2** | 2048 | -1.15 | -2.70; 0.42 |
| Model 1: adjusted for age, (sex); model 2: adjusted for age, (sex), systolic blood pressure, HDL cholesterol, LDL cholesterol, diabetes, anti-hypertensive medication, lipid-lowering medication, BMI and current smoking. | | | |
