# Supplementary material for: Socioeconomic position is associated with N-terminal pro-brain natriuretic peptide (NT-proBNP)—Results of the population-based Heinz Nixdorf Recall study
Source: PLoS One. 2021 Aug 20;16(8):e0255786. doi: 10.1371/journal.pone.0255786 (PMC8378685; doi:10.1371/journal.pone.0255786)
Supplement: S8 Table — (DOCX) [file pone.0255786.s008.docx]

**S8 Table.** Effect size estimates as percentage change in NT-proBNP and 95% confidence intervals (95%-CI) using sex-specific income quartiles (4^th^ as reference) in the analysis population after excluding participants with prevalent coronary heart disease and stroke and stratified by sex.

| **All** | | | |
| --- | --- | --- | --- |
| **Model** | **N** | **%-Change** | **95%-CI** |
| **Model 1** | 3898 |  |  |
| 3. Quartile |  | 6.80 | -0.75; 14.93 |
| 2. Quartile |  | 9.51 | 1.61; 18.03 |
| 1. Quartile |  | 9.73 | 1.76; 18.33 |
| **Model 2** | 3636 |  |  |
| 3. Quartile |  | 6.80 | -0.80; 14.98 |
| 2. Quartile |  | 8.63 | 0.71; 17.17 |
| 1. Quartile |  | 8.34 | 0.31; 17.00 |
| **Men** | | | |
| **Model** | **N** | **%-Difference** | **95%-CI** |
| **Model 1** | 1905 |  |  |
| 3. Quartile |  | 16.19 | 4.22; 29.53 |
| 2. Quartile |  | 18.27 | 6.22; 31.67 |
| 1. Quartile |  | 18.04 | 5.91; 31.57 |
| **Model 2** | 1761 |  |  |
| 3. Quartile |  | 12.41 | 0.70; 25.49 |
| 2. Quartile |  | 16.10 | 4.16; 29.41 |
| 1. Quartile |  | 13.60 | 1.68; 26.92 |
| **Women** | | | |
| **Model** | **N** | **%-Difference** | **95%-CI** |
| **Model 1** | 1993 |  |  |
| 3. Quartile |  | -2.09 | -11.28; 8.05 |
| 2. Quartile |  | 1.35 | -8.62; 12.41 |
| 1. Quartile |  | 2.09 | -7.99; 13.26 |
| **Model 2** | 1874 |  |  |
| 3. Quartile |  | 0.93 | -8.59; 11.44 |
| 2. Quartile |  | 1.69 | -8.41; 12.90 |
| 1. Quartile |  | 3.52 | -6.87; 15.07 |
| Model 1: adjusted for age, (sex); model 2: adjusted for age, (sex), systolic blood pressure, HDL cholesterol, LDL cholesterol, diabetes, anti-hypertensive medication, lipid-lowering medication, BMI and current smoking. | | | |
