# Supplementary material for: Socioeconomic position is associated with N-terminal pro-brain natriuretic peptide (NT-proBNP)—Results of the population-based Heinz Nixdorf Recall study
Source: PLoS One. 2021 Aug 20;16(8):e0255786. doi: 10.1371/journal.pone.0255786 (PMC8378685; doi:10.1371/journal.pone.0255786)
Supplement: S9 Table — (DOCX) [file pone.0255786.s009.docx]

**S9 Table.** Effect size estimates as percentage change in NT-proBNP and 95% confidence intervals (95%-CI) using education categories (≥18 years of education as reference) in the analysis population after excluding participants with prevalent coronary heart disease and stroke and stratified by sex.

| **All** | | | |
| --- | --- | --- | --- |
| **Model** | **N** | **%-Change** | **95%-CI** |
| **Model 1** | 4160 |  |  |
| 14–17 years |  | 8.21 | -1.39; 18;73 |
| 11–13 years |  | 9.47 | 0.61: 19.11 |
| ≤ 10 years |  | 9.20 | -2.28; 22.02 |
| **Model 2** | 3879 |  |  |
| 14–17 years |  | 6.67 | -2.89; 17.16 |
| 11–13 years |  | 8.19 | -0.75; 17.94 |
| ≤ 10 years |  | 8.06 | -3.58; 21.11 |
| **Men** | | | |
| **Model** | **N** | **%-Difference** | **95%-CI** |
| **Model 1** | 1978 |  |  |
| 14–17 years |  | 9.32 | -3.02; 23.21 |
| 11–13 years |  | 11.72 | -0.44; 25.35 |
| ≤ 10 years |  | 22.91 | 0.84; 49.80 |
| **Model 2** | 1830 |  |  |
| 14–17 years |  | 4.48 | -7.42; 17.92 |
| 11–13 years |  | 5.48 | -6.27; 18.71 |
| ≤ 10 years |  | 17.31 | -4.45; 44.04 |
| **Women** | | | |
| **Model** | **N** | **%-Difference** | **95%-CI** |
| **Model 1** | 2182 |  |  |
| 14–17 years |  | 3.49 | -11.08; 20.46 |
| 11–13 years |  | 4.83 | -7.54; 18.85 |
| ≤ 10 years |  | 7.33 | -7.34; 24.33 |
| **Model 2** | 2048 |  |  |
| 14–17 years |  | 5.32 | -9.73; 22.88 |
| 11–13 years |  | 8.18 | -4.84; 22.99 |
| ≤ 10 years |  | 10.98 | -4.55; 29.04 |
| Model 1: adjusted for age, (sex); model 2: adjusted for age, (sex), systolic blood pressure, HDL cholesterol, LDL cholesterol, diabetes, anti-hypertensive medication, lipid-lowering medication, BMI and current smoking. | | | |
